# On the Reality of Quantum Collapse and the Emergence of Space-Time

**DOI:** 10.3390/e21030323

**Published:** 2019-03-25

**Authors:** Andreas Schlatter

**Affiliations:** Burghaldeweg 2F, 5024 Küttigen, Switzerland; schlatter.a@bluewin.ch; Tel.: +41-(0)-79-870-77-75

**Keywords:** quantum measurement, quantum collapse, thermal time, Minkowski space, Einstein equations

## Abstract

We present a model, in which quantum-collapse is supposed to be real as a result of breaking unitary symmetry, and in which we can define a notion of “becoming”. We show how empirical space-time can emerge in this model, if duration is measured by light-clocks. The model opens a possible bridge between Quantum Physics and Relativity Theory and offers a new perspective on some long-standing open questions, both within and between the two theories.

## 1. Introduction

For a hundred years or more, the two main theories in physics, Relativity Theory and Quantum Mechanics, have had tremendous success. Yet there are tensions within the respective theories and in their relationship to each other, which have escaped a satisfactory explanation until today. These tensions have also prevented a unified view of the two theories by a more fundamental theory. There are, of course, candidate-theories, but none has found universal acceptance so far [1]. There is a much older debate, which concerns the question of the true nature of time. Is reality a place, where time, as we experience it, is a mere fiction and where past, present and future all coexist? Is this the reason why so many laws of nature are symmetric in time? Or is there really some kind of “becoming”, where the present is real, the past irrevocably gone and the future not yet here? Admittedly, the latter view only finds a minority of adherents among today’s physicists, whereas philosophers are more balanced. There is work in the foundations of physics [2,3,4,5,6,7] where the role of time in understanding reality and in possibly finding a bridge between the different views, is addressed. In [5] there is a kind of program, which tries to use the insights around the nature of time to bridge between Quantum Mechanics and Relativity Theory. In this paper we want to develop such a program in the spirit of [5] and in a fairly rigorous way. 

## 2. The Model

### 2.1. Quantum Mechanics 

The ansatz for the mathematical theory of quantum physics is to represent a measurable property of a physical system, called an observable, as a self-adjoint operator $A\in L\left(H_{\mathbb{C}}\right)$ in the space of linear operators over a state space $H_{\mathbb{C}}$, which carries the structure of a complex Hilbert space. The values, which this property can assume in an experiment, are the corresponding eigenvalues λ∈ℝ of A. Quantum theory assigns probabilities to these eigenvalues, which are then observed by experimentalists in repeated experiments on identically prepared systems. To do this, states are represented as unit-vectors $\vec{c}\in ,H_{\mathbb{C}},\;\|\vec{c}\|=1.$ They can be linearly expanded on the basis of orthonormal eigenstates of A, ${\left\{|e_{k}\rangle \right\}}_{k\in K}\subset H_{\mathbb{C}}$, $\vec{c}=\sum_{k\in K}^{}c_{k}|e_{k}\rangle ,c_{k}\in \mathbb{C}.$ A probability, $p_{k}$, is then assigned to the measurement of eigenvalue $\lambda_{k},\;k\in K,$ by:

$$p_{k}={\left|c_{k}\right|}^{2}.$$

This assignment is also known as the “Born-rule” [8]. Let there be a quantum system, represented by a vector ${\vec{c}}_{x,t}\in l_{{\mathbb{R}}^{4}}^{2}$:

(1) $${\vec{c}}_{x,t}=\left\{c_{x}\left(t\right)|c_{x}\left(t\right)\in \mathbb{C},\;x\in {\mathbb{R}}^{3},t\in \mathbb{R},\;\sum_{x}{\left|c_{x}\left(t\right)\right|}^{2}=1\right\}.$$

We use summation instead of integration to stress the analogy with finite-dimensions. This lack of mathematical precision will not harm the arguments in the paper. The $c_{x}\left(t\right)\in \mathbb{C}$ represent amplitudes for the probabilities $p_{x}\left(t\right)={\left|c_{x}\left(t\right)\right|}^{2}$ to detect the system in position $x\in {\mathbb{R}}^{3}$ at time-point t∈ℝ. The points $\left(t,x\right)\in \mathbb{R}\times {\mathbb{R}}^{3}\approx {\mathbb{R}}^{4}$ are considered as degrees of freedom. “Time”, t∈ℝ, does have the role of describing change under preservation of identity and is hence not the value of an observable, like position $x\in {\mathbb{R}}^{3}$ [9]. At this point the space ${\mathbb{R}}^{4}$ is just considered as a set and must not be confused with empirical space-time, where observed physical systems manifest themselves. It is just a space of arguments or degrees of freedom and has a priori no further structure. We will revert to that later. The system, represented by ${\vec{c}}_{x,t}$, carries (information) entropy:

(2) $$S_{{\vec{c}}_{x,t}}=-\underset{x}{\sum }{\left|c_{x}\left(t\right)\right|}^{2}log_{2}{\left|c_{x}\left(t\right)\right|}^{2}.$$

Because probability has to be preserved, change in $H_{\mathbb{C}}$ happens by unitary time-evolution, ${\vec{c}}_{x,0}\to {\vec{c}}_{x,t}$, and is described by means of unitary operators $U_{t}\in L\left(H_{\mathbb{C}}\right)$:

(3) $${\vec{c}}_{x,t}=U_{t}{\vec{c}}_{x,0}=e^{-\frac{i}{h}Ht}{\vec{c}}_{x,0},U_{t}^{-1}=U_{t}^{*}.$$

$H\in L\left(H_{\mathbb{C}}\right)$ is a positive-definite energy-operator, which we assume to be constant. Note that $S_{{\vec{c}}_{x,t}}=S_{{\vec{c}}_{x,0}}$. By a theorem in [10] we know that the minimal time span, $\Delta t_{min}$, needed until ${\vec{c}}_{x,0}$ evolves into an orthogonal state, ${\vec{c}}_{x,t}={\vec{c}}_{x,0}^{⊥}$, is

(4) $$\Delta t_{min}=\frac{h}{4\left(\bar{E}-E_{0}\right)},$$

with $\bar{E}=\langle {\vec{c}}_{x,0}|H|{\vec{c}}_{x,0}\rangle$, and $E_{0}$ denoting the lowest eigenvalue of H. In (4) we assume that the minimum is attained.

We mentioned already, that the role of “time” t is to describe change. We can define the probability, that after “time” t a system ${\vec{c}}_{x,0}$ has changed, by:

(5) $$P\left(t\right)=1-{\left|\langle {\vec{c}}_{x,t}|{\vec{c}}_{x,0}\rangle \right|}^{2}.$$

It holds that $P\left(\Delta t_{min}\right)=1$. Hence we call $\Delta t_{min}$ a “quantum of certainty”. Since for systems, which consist of a large number of subsystems, P(t)≈1, ∀t>0, “time” remains continuous, but can be counted in units of $\Delta t_{min}$:

(6) $$d\tau =\frac{1}{\Delta t_{min}}dt.$$

The symmetry of the unitary evolution (3) of ${\vec{c}}_{x,t}$ is broken by collapse, induced through a measurement interaction. In a collapse, as described by von Neumann, the state vector of a system is projected to an eigenvector of an observable, $\vec{c}\to |e_{k_{0}}\rangle ,\;k_{0}\in K,$ and a measurement produces the corresponding eigenvalue. In particular systems, represented by ${\vec{c}}_{x,t}$, attain a position $\left(t,x\right)\in {\mathbb{R}}^{4}$, which can be observed. We consider the collapse to be a real physical fact and the entropy (2), which arises by decoherence over an apparatus and is reduced by the projection, dissipates to the environment [11]. This is in contrast to a merely epistemic interpretation of von Neumann collapse, where the projection is purely an update of the knowledge of an observer. Collapse, introduced this way, is a key constituent of empirical space-time, which we define to be the set of observed systems in ${\mathbb{R}}^{4}$. It is the distance-relation between these observed systems, which will define a metric structure on ${\mathbb{R}}^{4}$. So what is the metric structure of empirical space-time?

### 2.2. Thermal Clocks

Let the system, represented by the vector ${\vec{c}}_{x,t}$, be embedded in a reservoir of “temperature” T, where we interpret temperature at this stage simply as the average energy per bit of information $k_{B}T=\frac{\partial E}{\partial S}$. By the second law a collapse triggers dissipation of an (minimal) average energy-amount to the environment [11]:

(7) $$\bar{E}=S_{\vec{c}}k_{B}T.$$

By (4) the quantum state of the environment $|\varepsilon_{0}\rangle$ turns by this interaction into an orthogonal state $|\varepsilon_{1}\rangle$, $\langle \varepsilon_{0}|\varepsilon_{1}\rangle =0,$ in a (minimal) time interval:

(8) $$\Delta t_{min}=\frac{h}{4k_{B}TS_{{\vec{c}}_{x,t}}}.$$

The assumption $E_{0}=0$ will be justified later in concrete cases. The collapse of a quantum-system thus leads to a definite “update” of its environment over a duration of $\Delta t_{min}$. We call a collapse and the corresponding update $|\varepsilon_{0}\rangle \to |\varepsilon_{1}\rangle$ an “event”. 

The duration (8) of an event is through $S_{{\vec{c}}_{x,t}}$ explicitly system-dependent. Very generally, we define a (natural) clock to be a process of a physical system, which uniquely defines a duration or period. We say that two clocks move in step, if there holds for their periods $\Delta t_{1}$, $\Delta t_{2}$ and a fixed α∈ℝ, α>0:

(9) $$\Delta t_{1}=\alpha \Delta t_{2}.$$

We call such a pair of clocks, by slight abuse of terminology, synchronous. Hence by setting $\alpha =S_{{\vec{c}}_{x,t}}$ there is modulo synchronization a universal thermal clock with period:

(10) $$\Theta =\frac{h}{4k_{B}T}.$$

Equation (6) turns into:

(11) $$d\tau =\frac{1}{\Theta }dt=\left(\frac{4}{h}k_{B}T\right)dt.$$

### 2.3. Light-Clocks

Let us consider the special case, where the update of an event is driven by the energy of a photon $\left(\bar{E}-E_{0}\right)=\frac{3}{2}h\nu -\frac{1}{2}h\nu =h\nu$ and hence $T=\frac{h\nu }{k_{B}}$. For the period we get (after normalization by a factor four):

(12) $$\Theta =\frac{1}{\nu }.$$

An event does not just define a duration (12), but also a spatial distance via the de Broglie-relation $\Delta x=\lambda =\frac{h}{p}$. We now chose a specific class of perspectives on empirical space-time. This class consists of those perspectives, in which free photons “move” (we will discuss the term “moves” later) in a time-interval of $\Delta t=\frac{1}{\nu }$ a distance of Δx=λ, such that:

(13) $$\frac{\Delta x}{\Delta t}=\lambda \Delta \nu =c.$$

In other words it is the class of perspectives, where time and space-units are chosen in such a way, that the speed of light is always c. By this choice and the resulting invariance of c under change of perspective, we introduce a (pseudo) metric structure on empirical space-time, which turns into Minkowski space-time $M^{4}.$ The line element is $ds^{2}=c^{2}dt^{2}-dx^{2}-dy^{2}-dz^{2}$, and the linear isometries are the Lorentz transformations Λ. These perspectives are also called inertial reference-frames. All observable quantities in empirical space-time have to be Lorentz-invariant, because they must be independent of the chosen perspective. The same holds for the laws, since else the resulting observable quantities are perspectival also. 

In this paper we describe events by a generic mathematical model (8), (10), (12). There is work in the foundations of quantum mechanics [12], where the collapse and the emission of a photon are specifically described by using the direct-action picture of QED [13]. All (quantum) field theories, which have a direct-action formulation [14], allow this approach, if massive matter-waves instead of photons are used to induce the updates. We will do corresponding calculations in our model in Section 4. Our approach can also be applied to Gravity, which does not have a direct-action formulation [14]. 

There are different intuitions around empirical space-time. Terms like: “a photon moves”, suggest that observable systems are embedded in $M^{4}$, which is supposed to be a kind of pre-existent vessel. We rather share the view in e.g., [4,5,15], that empirical space-time is actually being created by events. Every event creates a space-time interval ds, and the distance relations of observed systems become thus dynamical. Note, that independence of the update ds of the direction of 3-momentum causes the homogeneity of space. The probability-amplitudes ${\vec{c}}_{x,t}$ and their interactions in Hilbert space do not live in this empirical space-time [4]. This is a crucial point, for which we will gain further evidence in Section 3.2. 

## 3. Some Consequences

### 3.1. Relativistic Invariance

By the de-Broglie relation, Δx~hp, observable space-coordinates will not be defined at point-level. This is reflected in scattering experiments, where in-and outgoing free particles are observed in $M^{4}$, yet the interaction processes at the smallest scale are a black box. They are mathematically described in $M^{4}$ by approximation-terms of the S-matrix, but never observed in empirical space-time. This leads to entities like virtual photons, which do not fulfill the relativistic mass-shell constraint. They are not part of empirical space-time [4]. Virtual photons generate a force, which over multiple events leads to observable acceleration of systems in empirical space-time. The probability of an event is observable though, and therefore its expression has to be Lorentz-invariant. At the level of wave functions we may encounter violations of invariance [16]. We know that we don’t find invariant orthogonal position operators, or that the momentum eigenfunctions are non-local. Finally, collapse violates relativity and is hence not a process in empirical space-time either. It is also important to note that certain questions, like through which slit a photon passed, have a priori no answer in empirical space-time. The reason is, that without a measurement there simply exists no corresponding event. If we try to interpolate and construct seamless description in $M^{4}$ through hidden variables for instance, then these variables must forever be unobservable, i.e., truly “hidden”, or they violate relativity [5].

### 3.2. Time

Contrary to the time-parameter at the level of Hilbert space, which has a purely logical function and has a priori no direction, thermal-time, measured by thermal clocks, does have a direction because of the irreversibility of the underlying process. Collapse leads to the erasure of former states and the dissipation (7) is irreversible [11]. Therefore empirical space-time carries a time-orientation, which is not implied by the metric structure of $M^{4}$ alone. The dynamical nature ensures that the future is not yet, and the past is no more existent but unalterably fixed. This corresponds to our deep intuition of reality as an evolving “present”. For a consistent construction of the “transient now” within empirical space-time $M^{4}$, see e.g., [6,17]. The beginning of thermal-time was the first event and hence the beginning of empirical space-time. If the average temperature was very high, then by (7) a big amount of energy was dissipated to the environment, like a big bang. There is no need for a special low-entropy initial state to account for the direction of time [5]. By the Feynman-Stückelberg interpretation of anti-particles, it is also clear that they cannot last in empirical space-time. What about “time” at the level of Hilbert space $H_{\mathbb{C}}$? 

Probabilities are in fact the probabilities of collapse, which lives outside of empirical space-time. Yet, they can be observed by statistical experiments in labs within empirical space-time. Therefore they have to be Lorentz-invariant. If probabilities are to be independent of inertial reference frames, then, in particular, the probabilities of two space-like separated measurements have to be independent of their time-order. Let us consider the probabilities of two spin-measurements A,B∈{±1} on a pair of entangled photons in freely chosable directions a,b. We denote the outcomes by A=(A,a) and B=(B,b). In the sequel we follow an argument in [18,19], and the formulation is independent of the details of quantum theory. Assume that the measurement A happens before B and that there is no influence from the “future”, what we call “no retro-causality” (the notion of “causation” is intricate and we use the word simply to express the independence of correlations with future events). This means that events in the “future”, in particular B, have no impact on the probabilities of A. Denote by $\lambda_{A}$ all the variables, which could otherwise influence A independently of a. Since A happens first, and there is no retrocausality, we have:

(14) $$P_{A}\left(A|a,\;B,b,\lambda_{A}\right)=P_{A}\left(A|a,\lambda_{A}\right).$$

The analogous conclusion holds for a reference frame, where B happens before A:

(15) $$P_{B}\left(B|b,\;A,a,\lambda_{B}\right)=P_{B}\left(B|b,\lambda_{B}\right).$$

Independence of $P_{A/B}$ of time-order together with (14), (15) and $\lambda =\lambda_{A}\cup \lambda_{B}$, define a Bell-local model:

(16) $$P\left(AB|a,b,\lambda \right)=P_{A}\left(A|B,a,b,\lambda \right)\cdot P_{B}\left(B|a,b,\lambda \right)=P_{A}\left(A|a,\lambda \right)\cdot P_{B}\left(B|b,\lambda \right)$$

Such models contradict quantum physics theoretically [20] and experimentally [21]. As a consequence, there are either preferential reference-frames for probabilities, contrary to relativity, or we have to drop the assumption of no retro-causality. There has been renewed interest in the question of retro-causality [22,23,24], and its existence seems, not least given the above result, a sensible way within a realist interpretation of quantum physics to avoid serious tension with relativity. (There are other possibilities, like to renounce to the causal Markov property, which prevents the first factorization in (16), or to assume a kind of gigantic conspiracy, which prevents the free setting of a and b. These assumptions seem less plausible though. In case we don’t attest to any of the notions in quantum physics a reality and take a purely epistemic view, then the above arguments are obsolete, of course). It is, however, evident that the picture of empirical-space-time, which we introduced above, where the “present” dynamically emerges through events, cannot be made compatible with retro-causality, not even in its temporally non-local form [24]. To save the invariance of probabilities, there must be a realm outside of empirical space-time, where “time” is symmetric. The realm of amplitudes in Hilbert space and their laws can therefore not live in empirical space-time. The two-state vector formalism [2,25] and the transactional interpretation [3,4] both take the future-dependence explicitly into account. It is well known, that empirical space-time is protected from observable consequences of the, as we have seen, necessary non-localities in the Hilbert-space realm. As the protection serves the simple fact, that collapse is fundamentally probabilistic and no superluminal signals or influences can be sent.

## 4. The Structure of Space-Time

### 4.1. Minkowski Space 

The true power of the thermal-time concept becomes apparent, if we look at multiple events of interacting quantum systems. Multiple interactions manifest themselves in empirical space-time by acceleration. Systems with constant acceleration, a, can be expressed in $M^{4}$ by use of Rindler-coordinates. We chose a co-moving coordinate system, which is defined in the wedge, limited by |x|=t, and given by the transformations:

(17) x=ϱcosh(κϑ), t=ϱsinh(κϑ),      ϱ≥0, −∞<ϑ<∞.

The line-element is:

(18) $$ds^{2}={\left(\frac{\kappa \varrho }{c^{2}}\right)}^{2}c^{2}d\vartheta^{2}-d\rho^{2}-dy^{2}-dz^{2}.$$

Contrary to velocity, acceleration is not purely perspectival and cannot be transformed away by Lorentz transformations. But there is a local inertial reference-frame at t=0, where the system is momentarily at rest. Assume that in this local rest frame there is a heat bath of temperature T. (We assume that the heat bath is radiation and that $k_{B}T\approx {\bar{E}}_{\nu }$, for ν such that *h*$\nu \ll k_{B}T$. Hence $E_{0}=\frac{1}{2}h\nu \approx 0)$. Equation (11) turns into:

(19) $$d\tau =\frac{4}{h}k_{B}T\frac{c^{2}}{a}d\vartheta .$$

We want to gauge the thermal-clock (19) with the special clock defined by a matter-wave with rest-mass $m_{0}$, frequency ω=2πν and corresponding acceleration $\kappa_{\omega }$. In the respective rest-frame the matter-clock has a period:

(20) $$d\tau =\frac{4}{h}E_{\omega }\frac{c^{2}}{\kappa_{\omega }}d\vartheta .$$

By the de Broglie-relation there holds with $k=\left|\vec{k}\right|$:

(21) $$E_{\omega }^{2}=\hbar^{2}\omega^{2}=c^{2}\hbar^{2}k^{2}+m_{0}^{2}c^{4}.$$

Further with $u_{\omega }=\frac{\omega }{k}$ denoting the phase velocity of the matter-wave and $v_{\omega }=c^{2}\frac{k}{\omega }$ the group velocity, we have:

(22) $$\kappa_{\omega }=2\pi \cdot u_{\omega }\cdot \omega .$$

By (22) Equation (20) turns into:

(23) $$d\tau =\frac{4}{h}\hbar \omega \frac{c^{2}k}{\omega^{2}}d\vartheta =\frac{c^{2}k}{\pi^{2}\omega }d\theta =\frac{v_{\omega }}{\pi^{2}}d\vartheta .$$

The gauge-equation is therefore:

(24) $$\frac{4}{h}k_{B}T\frac{c^{2}}{a}=\frac{v_{\omega }}{\pi^{2}}.$$

For the temperature T there consequently holds:

(25) $$T=T_{a,\omega }=\frac{\hbar av_{\omega }}{2\pi k_{B}c^{2}}.$$

Equation (25) is a generalized Davies-Unruh temperature and is perspectival. The de Broglie matter-waves are fundamentally relativistic entities [26,27], and the two velocities $u_{\omega },v_{\omega }$ represent the inclination of the space-and time axis, respectively. If we chose a massless (no rest-mass) wave, we are in the invariant situation, where $v_{\omega }=u_{\omega }=c$ and Equation (25) turns into the well known Davies-Unruh formula:

(26) $$T_{a}=\frac{\hbar a}{2\pi k_{B}c}.$$

As mentioned above, Equation (25) can be of importance, if the update of empirical space-time, as part of an event, happens by massive gauge bosons. Equations (24) and (25) also allow to apply our approach to Gravity (the general power of Equation (24) is demonstrated in [28]). 

### 4.2. Gravity 

Gravity has so far resisted a quantum formulation, which would find universal acceptance [1]. There have been various proposals over time, which we cannot honor in this paper. In a nutshell the crux is, that by the equivalence principle one counterpart of the interaction seems to be the metric field of space-time. This leads to highly non-linear behavior of the metric components, which is hard to reconcile with the linear structure in Hilbert space. In our model we assume that a possible gravitational quantum-interaction becomes manifest after multiple events by an acceleration in empirical space-time, and we do not go into the specifics of a possible interaction. Our results, however, will lead us to some thoughts about it in the next Section 4.3. Let us consider gravitational acceleration in the Newtonian limit at distance R of a mass M at relative rest. With the gravitational constant, G, we have:

$$g_{R}=\frac{GM}{R^{2}}.$$

If we gauge duration with light clocks, we need two events to write down the left hand side of Equation (24), since $g_{R}$ is, strictly speaking, not constant. We get:

(27) $$\frac{4}{h}k_{B}T_{g_{R}}\frac{c}{g_{R}}=\frac{2}{\pi^{2}}.$$

An alternative view could be to gauge with a hypothetical graviton 𝓰. Since this particle is supposed to have zero mass and spin two, we may assume that $E_{𝓰}=2E_{\gamma }$ and also derive Equation (27). Note in contrast to Equation (24), the factor two on the right hand side of Equation (27). With $E=Mc^{2}$, $l_{P}=\sqrt{\frac{G\hbar }{c^{3}}}$, and $A_{R}=4\pi R^{2}$ we derive from (27):

(28) $$k_{B}T_{g_{R}}A_{R}=4l_{P}^{2}Mc^{2}=4l_{P}^{2}E.$$

By using (26) we arrive at:

(29) $$g_{R}A_{R}=\frac{8\pi G}{c^{4}}E.$$

For the coupling constant, $\alpha_{G}=\frac{8\pi G}{c^{4}}$, we also have the less familiar notation $\alpha_{G}=\frac{8\pi l_{P}^{2}}{\hbar c}$, which we will use later. Equation (29), which holds on a spatial hyper-surface, can be generalized in covariant fashion to four dimensions, if we suppose the metric to be static. We repeat the derivation in [28], following the one in [29]. We consider an asymptotically flat, static background with global time-like Killing vector field $\xi^{a}$. The generalization of Newton’s potential φ can be defined by:

(30) $$\varphi =\frac{1}{2}log\left(-\xi^{a}\xi_{a}\right).$$

The exponential $e^{\varphi }$ is the red-shift factor, which defines a foliation of space-time in space-like surfaces S of constant redshift. In this set-up a particle on the corresponding Killing world-line (e.g., at rest if $\xi =\partial_{t}$) will have a four-acceleration perpendicular to S given by:

(31) $$a^{b}=-\nabla^{b}\varphi .$$

The left hand side of (29) formally turns into the more general expression:

(32) $$g_{R}A_{R}\to \overset{}{\underset{S}{\int }}e^{\varphi }\nabla \varphi \cdot dA.$$

Formula (32) is (modulo constants) exactly the expression for the Komar-mass M, and we indeed get, by accounting for the constants, the equivalent equation to (29):

(33) $$\frac{1}{2}\overset{}{\underset{S}{\int }}e^{\varphi }\nabla \varphi \cdot dA=\frac{4\pi G}{c^{2}}\cdot M=\frac{4\pi G}{c^{4}}E.$$

Re-expressed in terms of the Killing vector $\xi^{a}$ there holds:

(34) $$\overset{}{\underset{S}{\int }}dx^{a}\wedge dx^{b}\in_{abcd}\nabla^{c}\xi^{d}=\frac{8\pi G}{c^{4}}E.$$

By Stokes theorem and the identity $\nabla^{a}\nabla_{a}\xi^{b}=-R_{a}^{b}\xi^{a}$ (34) turns into:

(35) $$\overset{}{\underset{\Sigma }{\int }}R_{ab}n^{a}\xi^{b}dV=\frac{8\pi G}{c^{4}}E,$$

where Σ is a volume bounded by S. Since by (35) the Ricci tensor $R_{ab}$ equals zero in a massless region, relation (35) holds for any boundary surface S of Σ, as long as Σ comprises all the matter. By writing the right hand side as an appropriate integral over components of the energy-stress tensor $T_{ab}$ of matter we get:

(36) $$\overset{}{\underset{\Sigma }{\int }}R_{ab}n^{a}\xi^{b}dV=\frac{8\pi G}{c^{4}}\overset{}{\underset{\Sigma }{\int }}\left(T_{ab}-\frac{1}{2}Tg_{ab}\right)n^{a}\xi^{b}dV.$$

Finally, by considering a small, almost flat space-time and imposing that, if matter m crosses the screen, then the Komar integral changes by that amount, (36) can be shown to hold for all (approximate) Killing vectors $\xi^{a}$ and screens S with normal vector $n^{a}$. Therefore:

(37) $$R_{ab}=\frac{8\pi G}{c^{4}}\left(T_{ab}-\frac{1}{2}Tg_{ab}\right).$$

A similar approach was taken in [30] by using null-screens. Of course, the derivation of Equation (37) bases on the a priori existence of a space-time symmetry (time-like Killing field), which allows the local definition and conservation of non-gravitational energy-momentum and hence of mass (we do not enter into the discussion oft the fact that, strictly speaking, also the affine connection $\Gamma_{g}$ could be freely chosen). The well-known difficulty to do the same for gravitational energy, even in presence of the symmetry, invokes some thoughts on the nature of the gravitational field. This even the more so, that the possibility of transformation of one energy-form into another sheds doubt on the definability of non-gravitational energy also [31,32].

### 4.3. Some Thoughts on Gravity 

In this paragraph we want to add some thoughts on gravity, inspired by the above results, which are more speculative in nature. Whatever the right gravitational interaction is, we have seen that the metric structure of space-time (37) can emerge from gauging the duration of multiple events in empirical space-time with light clocks. The special form of the gravitational acceleration, namely its independence of any probe-mass (weak equivalence principle) and the inverse-square dependence on spatial distance in the Newtonian limit, is fundamental to the result. Note that, if there were only one type of charged particle, the electron for instance with mass $m_{e}$, then the identical derivation as above would work with the Coulomb-acceleration:

(38) $$a_{R}=\frac{e^{2}}{4\varepsilon_{0}m_{e}R^{2}}.$$

This would lead to a coupling-constant $\alpha_{C}=\frac{8\pi \lambda_{C}^{2}}{\hbar c}$, instead of $\alpha_{G}=\frac{8\pi l_{P}^{2}}{\hbar c}$, where $\lambda_{C}=\frac{h}{m_{e}c}$ is the Compton wavelength [33]. 

In the last few decades there has been a series of approaches to explain gravity as an emergent phenomenon [28,29,34,35,36]. We cannot give individual credit to these works here, and for a recent overview see e.g., [37]. Our approach differs in two main points. Firstly, already empirical space-time at the level of Minkowski space $M^{4}$ is emergent. More precisely, it is the metric structure, which emerges as a result of events and the choice of light clocks to measure duration (13). From there it only needs the gravitational acceleration in the Newtonian limit, to derive the dynamics of the metric structure and hence general relativity. Secondly, the irreversibility of events and their interpretation as “becoming” leads to the existence of two realms. On the one hand the realm of Hilbert space, where time is necessarily symmetric, and on the other hand the growing block-world of empirical space-time [5]. The concept of a dynamical space-time, which emerges through events from an abstract realm, points to the possibility that the metric-field is not the counterpart in a quantum-interaction of gravity but really an emerging relational structure on empirical space-time. (This idea follows the tradition of Leibniz and Mach). The gravitational interaction would then happen between material systems outside of empirical space-time, totally in line with the other interactions [38], or it could be the result of already known, but not yet calculated quantum effects, as R. Feynman suggests in his lectures on Gravitation (2003) Section 1.5 [39]. The difficulty to define energy and its conservation in empirical space-time, whether gravitational or non-gravitational, might then reflect that it is not the metric field, which carries energy, but some other fields in Hilbert space. It is, on the other hand, to be expected that the emerging metric structure does somehow reflect energy of the fundamental fields, as the example of gravitational waves proves. A satisfactory mathematical definition within empirical space-time seems, however, only possible in special cases and in a linearized approximation [30]. Nevertheless the recent LIGO experiments have been very accurately predicted by the mathematics of general relativity. The metric structure must consequently reflect energy for all forms of acceleration, as the physical length contraction, which is considered to be an effect in special relativity, shows [40]. By the same token, the fact that there is the Planck length $l_{P}$, below which a massive particle would be hidden by its own horizon, might just be a fact of the emergent geometry of empirical space-time and need not be the result of a quantization of space-time itself. The corresponding Planck-time is then given by tP=lPc, in line with our model (13). We get the Planck mass $m_{P}$, if the Compton wavelength equals the Planck length, $\lambda_{m_{P}}=l_{P}$, and hence $\alpha_{C}=\alpha_{G}$. This in turn allows the definition of the gravitational constant by:

(39) $$G=\frac{\hbar c}{m_{P}^{2}}.$$

So there is a connection between electrodynamics and gravity at the Planck scale. Maybe it is just the other way round, and there is a fundamental quantum of mass $m_{P}$, acting like charge in a field theory, from which the other quantities like $l_{P}$, or G follow. In this context it also seems natural, that in the mathematical formulation of quantum-interactions in $M^{4}$, without empirical backing at the smallest scale, integration is cut at $l_{P}$ and some infinities are avoided. 

## 5. Conclusions

We have given arguments for the existence of two realms, one of probability amplitudes in abstract Hilbert space, which carries a symmetric temporal structure, and one, which emerges by events as a result of breaking unitary symmetry. Events create empirical space-time. Gauging the duration of one or multiple events by light clocks, directly leads to a relativistic and time-directed metric structure of empirical space-time. The dual picture of reality adds to a better understanding of our intuitions about time and the treatment of it in physics. The choice of light clocks is maybe the result of our human condition to mainly perceive the world by means of light. The answer to the centuries-old question, whether reality is static, as taught by Parmenides, or dynamic in the tradition of Heraklit, would then be: it is both and there is an interplay between the two sides of the medal. 
